# The Perceptions of Children and Adolescents with Cancer Regarding Nurses’ Communication Behaviors during Needle Procedures

**DOI:** 10.3390/ijerph19159372

**Published:** 2022-07-30

**Authors:** Encarna Gómez-Gamboa, Olga Rodrigo-Pedrosa, Marta San-Millán, Maria Angeles Saz-Roy, Anna Negre-Loscertales, Montserrat Puig-Llobet

**Affiliations:** 1 Advanced Practice Nurse, Sant Joan de Déu Maternity and Chidren’s Hospital, 08950 Esplugues de Llobregat, Spain; egomez@sjdhospitalbarcelona.org (E.G.-G.); anegre@sjdhospitalbarcelona.org (A.N.-L.); 2 ESIMar (Mar Nursing School), Universitat Pompeu Fabra-Affiliated, 08003 Barcelona, Spain; 3 Embriology and Neuroscience Research Group (NEOMA), Medical Sciences Department, Clinical Anatomy, Faculty of Medicine, University of Girona, 17071 Girona, Spain; m.sanmillan@udg.edu; 4 EUSES University School of Health and Sports, University of Girona, 17190 Salt, Spain; 5 School of Nursing, University of Barcelona, 08907 Hospitalet de Llobregat, Spain; msazroy@ub.edu (M.A.S.-R.); monpuigllob@ub.edu (M.P.-L.)

**Keywords:** adolescent, cancer, child, communication barriers, compassion fatigue, qualitative approach, needle procedures, oncology nursing, working environment

## Abstract

**Background:** Communicating with children and adolescents with cancer during a needle procedure can prove challenging for healthcare professionals. **Objective:** Our aim was to explore the perceptions of children and adolescents with cancer regarding communication with nurses during needle procedures. **Method:** Thus was a qualitative phenomenological study. Data were gathered through seven in-depth interviews with a convenience sample of children and adolescents with cancer. Data were analyzed using a grounded theory approach to identify themes in the participants’ narratives. **Results:** The analysis revealed three themes describing participants’ experience: (1) nurses need to explain clearly what they are going to do while also allowing children to express their emotions without feeling coerced; (2) nurses need to be honest and approachable and relate to children as active participants in the treatment process; and (3) it is distressing to hear other children who are undergoing a needle procedure cry out in pain. Further application of the constant comparison method yielded a core theme: (4) the pressures faced by oncology nurses lead them to focus on the technical side of procedures at the expense of their young patients’ communication needs. **Conclusions:** We suggest that hospital managers need to ensure that oncology nurses have sufficient training in communication skills and are confident in their ability to respect and respond to the communication preferences and needs of patients.

## 1. Introduction

Children and adolescents with cancer have to deal with a life-threatening diagnosis, intensive treatment, and invasive procedures [1]. The latter are often painful and frightening [2], and they are regarded by many young patients as the most stressful aspect of their illness [3]. Effective communication between children and nurses is therefore important to improve satisfaction with care and to facilitate the best possible outcomes [4]. However, given that these patients will be at different developmental stages, and that decision-making power lies ultimately with their parents, communication can be particularly challenging for professionals [5], especially when performing invasive procedures [6].

The treatment of childhood cancer is a long process that invariably involves many painful procedures which can impact patients on a daily basis [7] producing significant suffering [8]. Venipuncture, for instance, is a common source of pain among hospitalized children [9] and is associated with considerable distress [10]. Children are generally less capable than adults of understanding the reason for these procedures, how long they might take, or how much discomfort they may experience [11], although their memory for stressful events of this kind tends to be fairly accurate [12]. However, a lack of understanding about an invasive procedure [13] and distress while it is being performed may exaggerate negative memories in children, leading to even greater distress when they have to undergo similar procedures in the future [12,14].

Communication difficulties, the stress associated with invasive procedures, and being cut-off from family and social life due to long periods of hospitalization are all factors that can impact children’s wellbeing [14], with studies showing that the negative effects may persist for years after cancer treatment ends [15]. Post-traumatic symptoms such as nightmares, flashbacks, constricted affect, anger, and an exaggerated response have been reported among adult survivors of childhood cancer [16], and they are related to the experience of invasive procedures in particular [17]. Being able to talk about these experiences and incorporate them into a coherent personal story can help patients cope with the emotional impact of their experiences [18], and hence it is important that health professionals are able to facilitate this at the same time as managing pain and distress through appropriate techniques [7]. Good pain management is especially important in the pediatric setting [9] and requires adequate knowledge and training among nurses [19] including as regards effective communication skills [20].

Various studies have examined communication in pediatric oncology from the perspective of parents or health professionals [21,22,23,24,25], although few have gathered the views of children themselves [26,27], especially in relation to their experience of needle procedures. This is an important gap, since a better understanding of children’s communication preferences could help to ensure they receive the kind of support they need [28], which in turn could improve their psychological wellbeing [14].

The impact of health professionals’ communication behaviors on patients has been identified as a priority target for research [29] insofar as there is a need to develop effective interventions for managing treatment-related stress, especially among children [30]. Accordingly, and given that the hospital care and treatment of children and adolescents with cancer is primarily administered by nurses, it is important to gather the views of these young patients regarding communication with nurses during potentially distressing procedures.

### Aims

The aim of this study was to explore the perceptions of children and adolescents with cancer regarding communication with nurses during needle procedures.

## 2. Materials and Methods

### 2.1. Study Design

Given that our goal was to describe and understand the lived experience of participants and to identify key concepts in their personal narratives, we used an inductive, phenomenological approach [31] for this qualitative study.

### 2.2. Participants

We recruited a convenience sample of children and adolescents with cancer whose treatment required a needle procedure. With the aim of achieving maximum variation in terms of participants and contexts [32], we selected both male and female patients of different ages and sought to include different types of cancer (see Table 1). Recruitment was carried out in the pediatric oncology service of a reference center for the treatment of childhood cancer in Spain. Each year, the unit provides treatment to around 2000 children and adolescents with various types of cancer. Recruitment of participants continued until theoretical saturation was reached and interviews yielded no new information.

### 2.3. Data Gathering

Data were gathered through eight in-depth individual interviews. Following the recommendations of Seidman [32], these involved the sequential exploration of three topics so as to facilitate expression of the participant’s lived experience and to situate it in a socio-historical context (see Table 2). Specifically, we began by asking participants to talk generally about their experience to date of needle procedures, before exploring in more detail their current experience as an in-patient, including as regards communication with nurses. We then asked them to talk about what it meant to them that they had to undergo routine needle procedures as part of their treatment. Interviews lasted between 30 and 50 min.

Potential participants were identified through a member of the research team who worked as a clinical nurse in a pediatric oncology unit. Initial contact was made by telephone, informing the children’s parents about the nature of the study and requesting their participation. Those who agreed were then invited for an interview at a time and place of their choosing, with informed assent (children) and informed consent (parents) being obtained prior to any data collection. All interviews were conducted by a member of the research team who was a nurse specialist in pediatrics and had no previous contact with participants and who had specific training in the psychological care of children and adolescents. In accordance with the personal preference of all of the children and adolescents who took part in the study, a parent (in all cases, the mother) was also present during the interview.

### 2.4. Data Analysis

All interviews were audiotaped and transcribed verbatim. The transcripts were then analyzed using the constant comparative method, an approach informed by grounded theory, in order to extract as much information as possible from the interviews. As recommended by Strauss and Corbin [33] we began with the open (substantive) coding of data, linking fragments of the participants’ discourse to coding labels. Having identified the most frequently occurring codes (focused coding), we then drew up a provisional set of categories and described their properties and dimensions (axial coding). By applying this coding paradigm throughout the analytic process, we were ultimately able, as part of the process of selective coding, to establish relationships between the categories. This was then complemented by a literature review in order to contextualize our findings and to develop the theoretical narrative that is presented in the Results Section below. As further support for this narrative and the analytic process, we also generated a series of memos (code, theoretical, operational, and bibliographic) [34]. The data were analyzed independently by two members of the research team data analysis was performed using ATLAS.ti 7.1 GmbH (Berlin, German).

### 2.5. Rigor

We employed a number of strategies to ensure validity and reliability. The use of work standards and the ATLAS.ti software helped to ensure a systematic approach throughout the analytic process, which was also subject to both internal and external audit. As for the credibility of our findings, these are illustrated throughout with verbatim quotations from the interview transcripts. Regarding transferability, we describe the sampling context and acknowledge that the results may not be generalizable to other populations or settings. Finally, all of the researchers involved in analyzing the data kept a reflexive journal in order to ensure the confirmability of results.

### 2.6. Ethical Consideration

The study was conducted in accordance with the principles of the Declaration of Helsinki and was approved by the Clinical Research Ethics Committee of Fundació Sant Joan de Déu (PIC-63-17). As already noted, informed assent and informed consent was obtained in all cases prior to data collection. A data protection protocol was established in accordance with current legislation and requirements in our country.

## 3. Results

The analysis of interviews revealed three themes in participants’ accounts of communication with nurses during a needle procedure: (1) nurses need to explain clearly what they are going to do, while also allowing children to express their emotions without feeling coerced; (2) nurses need to be honest and approachable and relate to children as active participants in the treatment process; and (3) it is distressing to hear other children who are undergoing a needle procedure cry out in pain.

Further application of the constant comparative method revealed a core theme that underpins interpersonal communication between nurses and pediatric oncology patients: (4) the demands of the working environment mean that nurses are focused on the technical side of procedures, and thus they may fail to consider the communication needs of young patients.

### 3.1. Nurses Need to Explain Clearly What They Are Going to Do, While Also Allowing Children to Express Their Emotions without Feeling Coerced

The young patients we interviewed considered that nurses needed to improve their communication when performing a needle procedure, explaining things clearly and, above all, in a positive tone [28].

*With children, especially those of my age, I think you need to be gentle, to talk to them gently. “OK, so this is what we’re going to do*

*(Participant 1)*

*If someone comes and they’re in a bad mood when they tell you things. How can I put it… I think they should be more tactful in how they say things. I think it’s important*

*(Participant 4)*

*A happy mood, that’s what they need to show*

*(Participant 3)*

*A lot of them already do it, but if they talk to you in a more positive, happy way, well all the better*

*(Participant 4)*

*Sometimes they say things really quickly, and sometimes they mumble. They need to speak up, because if they mumble we don’t get what they’re saying*

*(Participant 7)*

*Sometimes they speak in Catalan and I don’t understand. So then I tell them to speak Spanish so that I can understand them*

*(Participant 7)*

Having the opportunity to express their feelings related to the pain, stress and fear associated with needle procedures was also important to the young patients we interviewed.

*Well, because they shouted at you, they shouted. Some people just don’t understand the pain a child can feel*

*(Participant 1)*

*They say to you: “We’re going to take some blood, and don’t cry because it’ll be quick*

*(Participant 7)*

*It’s intimidating when four or five people come and tell you what you have to do*

*(Participant 1)*

*Yes, with the new staff, because then they learn. Because they know that I won’t*

*(Participant 3)*

This suggests that what is lacking here is an encounter characterized by assertive communication, whereby nurses are able to be clear and direct while simultaneously respecting the rights and needs of children and adolescents to express what they think and feel [35]. As a style of communication, assertiveness also underpins the establishment of a therapeutic relationship [36].

### 3.2. Nurses Need to Be Honest and Approachable and Relate to Children as Active Participants in the Treatment Process

In the view of our interviewees, nurses need to be honest Smith et al. [28] and approachable, which also means engaging with patients during treatment so as to build a relationship of trust.

*You trust them more than if they say, oh, it’s not going to hurt, or whatever*

*(Participant 4)*

*Someone like that, you trust them more than someone who is cold or distant and who tells you it won’t hurt*

*(Participant 4)*

*I like them to be straight with me. For example, in this hospital they tell it like it is. They don’t say one thing to your mother and something different to you*

*(Participant 4)*

*For example, you ask if it’s going to hurt and they say no, but then it really hurts. If it’s going to hurt, then say so, and then the next time try to do it better and I won’t be so nervous*

*(Participant 7)*

*I was crying. I said I couldn’t take it any more. Don’t you remember [child turns to his mother] that they said they were going to give me a prize, but they didn’t*

*(Participant 7)*

*All the nurses are lovely. If it’s the first time we’ve met, then they’ll say “My name’s so and so, and I’ll be here for a few days”. I’ve got to know almost all the nurses on the ward!*

*(Participant 6)*

*I like them to tell me what they’re going to do*

*(Participant 5)*

*It depends on the nurse. Sometimes they do talk to you when they’re doing something to you. But normally they’re talking among themselves*

*(Participant 1)*

*It depends on the nurse, but normally no. It’s more about getting the job done*

*(Participant 3)*

*They do talk to the younger children, so that they concentrate*

*(Participant 3)*

*They don’t always ask me if the injection hurt*

*(Participant 3)*

*Sometimes they ask me if the injection hurt*

*(Participant 4)*

A study by Ruhe et al. [37] likewise found that pediatric oncology patients felt dissatisfied when they were not treated as participants in the care process, and that this made it more difficult for them to understand their illness. Various studies have found that children are often marginalized in the hospital environment, insofar as professionals communicate primarily with parents [38]. Communication with children about their care is obviously a challenge, given that nurses need to consider the child’s age, cognitive ability, behaviors, and physical and psychological condition, as well as the illness stage and response to treatment [39]. This is further complicated in the oncology setting, as nurses may inhibit communication with patients if they find it hard to deal with emotionally charged topics such as death and dying [40]. Other reasons for ineffective communication include feelings of despair, difficulty coping with stress, lack of skills, and a high workload [40].

### 3.3. It Is Distressing to Hear Other Children Who Are Undergoing a Needle Procedure Cry Out in Pain

Hearing other children cry as they undergo a needle procedure was something that our participants found distressing.

*I like the door shut, because you can hear people in other rooms, people crying, for example. It doesn’t help*

*(Participant 1)*

*I never like to hear other children crying, because I put myself in their shoes*

*(Participant 2)*

*It bothers me. Things are quiet, but then you hear this “ahhh” [a child crying]. It’s upsetting. But then you tell yourself, well, it’s what you’d expect. You’d expect this in a hospital*

*(Participant 3)*

*Well of course, when you hear other children crying or whatever, you think to yourself ‘God, I bet it’s going to hurt!’*

*(Participant 4)*

*When it’s the little kids’ turn, when they see the needle and they begin to scream, ‘ahhh!. On the one hand, you think, ‘poor things’, but they’re also a bit annoying*

*(Participant 6)*

*It upsets me to hear them cry*

*(Participant 5)*

*I feel sorry for those who cry*

*(Participant 8)*

A well-designed hospital environment, which includes provision for privacy, can contribute to the psychological wellbeing of patients [41]. Privacy is regarded by Altman [42] as a process of selective control over social interaction, and for Shepley [43] it is more important than the interaction itself. For parents and children, this also implies the possibility of auditory privacy [43], which means that communal areas of hospitals, such as those where invasive procedures are performed, need to be designed in such a way that one cannot hear other people’s conversations with health professionals [42].

Our interviewees found it both upsetting and annoying to hear other children cry, and it also reminded them of their own pain during needle procedures. The latter may have its basis in the mirror-neuron system, which is activated not only during individual action but also when observing a similar action in others [44]. This system of neural circuits enables us to perceive the emotions of others [44], as if we ourselves were experiencing them. This is necessary for our survival as it allows us to show empathy, which is fundamental to social interaction [45]. However, it also means that when perceiving pain or distress in others, we may also experience the associated physiological activation [46].

### 3.4. The Demands of the Working Environment Mean That Nurses Are Focused on the Technical Side of Procedures, and Thus They May Fail to Consider the Communication Needs of Young Patients

The children and adolescents we interviewed felt that nurses needed to consider their need for communication, and not focus solely on the technical side of procedures.

*I’d like them to devote more time to me. It’d be nice if they didn’t just talk to each other but also to me*

*(Participant 1)*

*But I also understand, because I realize they have lots to do. So I guess they talk to you like that because they’ve got more important things to do. They’re in a hurry and all that*

*(Participant 1)*

*And I Would close my eyes and tell nurses to wait for me to ready*

*(Participant 8)*

This emphasis on technique was attributed by participants to a lack of time and boredom or weariness among nurses.

*So I closed my eyes and told the nurses to wait until I was ready*

*(Participant 5)*

*If someone comes and they’re in a bad mood when they tell you things*

*(Participant 4)*

*Because sometimes they seem bored*

*(Participant 7)*

This weariness or boredom that our participants referred to may suggest the presence of compassion fatigue among the nurses with whom they had contact.

*I’d like them to devote more time to me. It’d be nice if they didn’t just talk to each other but also to me*

*(Participant 1)*

*But I also understand, because I realize they have lots to do. So I guess they talk to you like that because they’ve got more important things to do. They’re in a hurry and all that*

*(Participant 1)*

*If someone comes and they’re in a bad mood when they tell you things*

*(Participant 4)*

*Because sometimes they seem bored*

*(Participant 7)*

Compassion fatigue is not uncommon among nurses who regularly care for patients with a life-threatening illness [46], and it can cause a pervasive decline in their desire, ability and energy to care for others [47]. The literature indicates that nurses working in oncology are more at risk of suffering compassion fatigue [48], although a high workload and a lack of support from colleagues and managers are also determining factors [49].

## 4. Discussion

In this study, we explored the perceptions of children and adolescents with cancer regarding communication with nurses, specifically when undergoing the needle procedures that form a routine part of their treatment. The analysis of interviews revealed a series of factors that appear to make communication difficult. The first has to do with a high workload, leading nurses to focus on the technical side of procedures at the expense of the young person’s need to communicate. The second factor relates to a possible lack of communication skills among nurses, insofar as the children and adolescents we interviewed wanted nurses to speak more clearly and honestly while also allowing them to express their own thoughts and feelings. Finally, fatigue among nurses may also make it difficult for them to pay due attention to the communication needs and preferences of pediatric oncology patients.

Regarding a heavy workload, this is known to be among the factors that, in the view of nurses, can undermine their ability to provide adequate patient care [50]. Poor staff allocation and ward management, along with a high patient-nurse ratio, can create an unhealthy working environment for nurses, leading to occupational stress, an increased likelihood of errors, and job dissatisfaction [51]. One of the ways in which nurses manage the pressures of their workload is by adopting a cold attitude and maintaining a professional distance, something which our interviewees interpreted as a lack of understanding, honesty, and positivity, all of which can undermine patient care [52]. This professional distance would also explain why our participants felt there was a greater emphasis on technique than on the person being treated. The automatization and standardization of care, which is often linked to high workload, can also lead to the depersonalization of care [53], which again is reflected in the experiences of our interviewees. Together with the published research, our findings highlight the need for nurse managers to put in place systems that enable workload to be closely monitored in these types of services, where patients’ needs, and therefore the required staffing ratio, are constantly changing. In this sense, the interviews in this study were carried out before the COVID-19 pandemic, specifically in the years 2018–2019 and, therefore, the situation in the health system may have worsened the overload of nursing work even more (something which we believe should be investigated).

Our analysis also suggested the need for improved communication skills among nurses. As other authors have found [28], the children and adolescents we interviewed wanted nurses to be clear and honest, while also respecting their right and need to express their own feelings. It seems, therefore, that what is lacking is the ability among some nurses to engage in assertive communication. The interpersonal relationship and effective communication with patients are key factors underpinning the quality of nursing care [54,55,56,57], and they are especially important in the pediatric setting since children are likely to find it harder than adults to cope with being hospitalized [58]. Communicating with oncology patients brings further challenges, and it is therefore important to provide nurses with adequate training, not least as confidence in one’s ability to communicate is vital to job satisfaction and stress management, and since ineffective communication leaves patients feeling dissatisfied with care [59]. Hospitals with haemato-oncology units therefore need to ensure that staff have the opportunity to develop the communication skills required to care for young patients with a potentially life-threatening illness.

Finally, the distress that nurses themselves experience when regularly caring for children with a severe and potentially life-threatening illness may, over time, lead to compassion fatigue [50], and this could further inhibit their ability to consider the communication needs of young patients during needle procedures. An exacerbating factor here is that nurses usually have little time to reflect on their practice [60], which is an important gap given the stress produced by a heavy workload and the responsibility of caring for these patients [50]. Continuous occupational stress of this kind [61] can lead to burnout in the form of emotional exhaustion, depersonalization and a sense of poor personal accomplishment [62]. Research suggests that as many as one in four nurses may experience burnout [63] and those working in pediatric oncology units are likely to be more exposed to specific risk factors such as direct contact with death and dying, the suffering of patients and families [64], an excessive workload [50]. Emotional exhaustion and depersonalization among health professionals may be key factors contributing to the presence of structural violence or cruelty in hospital settings [65], which in the experience of our interviewees was characterized by communication that was insufficiently clear and honest, a lack of auditory privacy and a failure on many occasions to respect and allow them to express their feelings. Despite conducting an extensive literature search, we found few studies that address the issue of structural violence in hospitals, especially in relation to children with life-threatening illnesses. This highlights the need for further research, and particularly participant observation studies, so as to determine the extent to which these behaviors are present within complex care settings. The present study contributes to this goal, insofar as the interviews with children and adolescents suggest that one of the ways in which oncology nurses cope with the pressures and responsibilities of their working environment is by providing dehumanized care, which paradoxically can end up diminishing their sense of personal accomplishment and increasing their likelihood of burnout.

## 5. Conclusions

Our analysis of interviews with children and adolescents with cancer regarding their experience of communication with nurses when undergoing routine needle procedures indicates that nurses often focus on the technical side of these procedures, at the expense of the young person’s communication needs. Being given the opportunity to express their own thoughts and feelings (without feeling coerced) was important for these young patients, and they wanted nurses to be clear and honest, relating to them as active participants in the treatment process. Privacy in the hospital setting is also important, since hearing other children cry as they undergo a needle procedure can be distressing.

The primary limitation of our study is that the research team was comprised solely of female professional nurses, and therefore any preconceived ideas they share could have influenced the analysis and interpretation of data. With the aim of limiting this potential bias, all researchers involved in analyzing the data were required to keep a reflexive journal, recording their decisions and the reasons for them, as well as reflecting on their personal values and interests. We also conducted an extensive literature review so as to compare our interpretation with previous findings and to support the theoretical narrative we present in the results section.

## Figures and Tables

**Table 1:** Characteristics of the sample.

Participant	Age	Sex	Diagnosis	Treatment Phase	Siblings	Parent Present
1	13 years old	Female	Acute lymphoblastic leukemia	Presence of post-treatment sequelae No history of relapse	None	Parents together
2	16 years old	Female	Acute lymphobastic leukemia	History of relapse	Has 12 year-old-brother	Parents together
3	14 years old	Female	Acute lymphoblastic leukemia	Bone marrow transplant	Has 19-year-old brother	Parents together
4	18 years old	Male	Acute- lymphoblastic leukemia	No history of relapse	None	Parents together
5	10 years-old	Male	Acute-lymphoblastic leukemia	History of relapse	None	Parents separated
6	10 years old	Male	Acute-lymphoblastic leukemia	No history of relapse	None	Parents together
7	9 years-old	Male	Acute- lymphoblastic leukemia	No history of relapse	Has 3-year-old brother	Parents together (but father currently in Venezuela)
8	13-years-old	Female	Brain tumour	No history of relapse	Has 15-year-old brother	Parents together

**Table 2:** Themes to be explored.

Themes to Be Explored	Purpose of Exploring the Theme
Theme 1: Tell me something about your illness since you first learned that you had cancer	*To provide a socio-historical context in which to understand the participant’s lived experience*
Theme 2: Tell me something about what it’s like for you when you have to undergo treatment that involves needle procedures	*To gather information about the participant’s lived experience in relation to the study topic*
Theme 3: Tell me something about what it means for you to undergo these kinds of procedures	*To gather information about the meaning of this experience for the participant.*

## Data Availability

Not applicable.
